# Obesity in secure hospital settings: Changes in BMI over time among a complete national cohort of forensic in-patients in Dundrum Hospital, Ireland

**DOI:** 10.1192/j.eurpsy.2023.218

**Published:** 2023-07-19

**Authors:** M. U. Iqbal, M. U. Waqar, B. Ogunnaike, H. G. Kennedy, M. Davoren

**Affiliations:** 1 Department of Forensic Psychiatry, National Forensic Mental Health Service, CMH Dundrum; 2 Dundrum Centre for Forensic Excellence, Trinity College Dublin, Dublin, Ireland

## Abstract

**Introduction:**

There are high rates of treatment resistant psychoses and medical complexity among patients in secure forensic hospitals (Basrak et al., BJPsych Open (2021) 7, e31,1-7). Patients with schizophrenia in secure settings have a lower life expectancy compared to community peers of approximately 16 years. Evidence suggests patients in secure settings often gain significant amounts of body weight during their in-patient stays, many of whom develop complex obesity presentations.

**Objectives:**

To ascertain changes in Body Mass Index (BMI) among patients in a secure forensic hospital setting over a 3.5 year period.

**Methods:**

A prospective longitudinal study of repeated measures of BMI for all (n=91) patients in a National Forensic Mental Health Service (CMH Dundrum, Dublin, Ireland). BMI was measured six-monthly, giving up to seven time points for each patient. Generalized Estimating Equations (GEE) analysis was conducted to ascertain changes in BMI over time. This study formed part of the DUNDRUM Forensic Redevelopment Evaluation Study (D-FOREST) (Davoren et al., BMJ Open (2022) 12(7): e058581).

**Results:**

A total of 91 patients were included in the study, mean age 33.46 years (SD 9.23). Mean length of stay was 8.09 years (SD 9.23). The most common diagnosis was schizophrenia (67%), followed by schizoaffective disorder (17.5%) and Autistic spectrum disorder (6.2%). Using GEE with BMI as the dependent variable, for the complete patient cohort, BMI changed significantly with diagnosis (Wald X^2^=5817.58, df=7, p<0.001). Those with severe mental illnesses (psychoses) had the highest BMI of the group, and BMI tended to increase over time (p=0.109). Among patients who were in the secure hospital for four years or less, their weight gain was significant over time (Wald X^2^ =10.0, df=1, p=0.002).

**Conclusions:**

We have shown high rates of obesity particularly in patients with psychoses and we have shown weight gain is significant during the first four years after admission to a national forensic service. This is a significant health concern and an area of unmet treatment need which is likely generalizable across secure hospitals in the EU.

**Disclosure of Interest:**

None Declared

